# Protective Effects of Different Molecular Weights of Purslane (*Portulaca oleracea* L.) Aqueous Extract on DSS-Induced Ulcerative Colitis in Mice

**DOI:** 10.3390/antiox12071400

**Published:** 2023-07-08

**Authors:** Ke Ning, Yameng Duan, Weiwei Tong, Yue Chen, Qinghui Zhang, Qiuhong Xie, Hongyu Xiang

**Affiliations:** 1 Key Laboratory for Molecular Enzymology and Engineering of Ministry of Education, School of Life Sciences, Jilin University, Changchun 130012, China; ningke20@mails.jlu.edu.cn (K.N.); duanym19@mails.jlu.edu.cn (Y.D.); tongww20@mails.jlu.edu.cn (W.T.); yuechen21@mails.jlu.edu.cn (Y.C.); qinghui20@mails.jlu.edu.cn (Q.Z.); 2 Institute of Changbai Mountain Resource and Health, Jilin University, Fusong 134504, China

**Keywords:** ulcerative colitis, macromolecular extract, purslane, oxidative stress, small molecular extract, gut microbiota, *Portulaca oleracea* L.

## Abstract

Purslane, a common wild vegetable, contains active substances with various biological functions. However, its effects have been under-investigated in ulcerative colitis (UC). Therefore, this study investigated the therapeutic effects of purslane macromolecular (POEM) and small molecular extracts (POES) on dextran sulfate sodium (DSS)-induced UC in mice. Membrane separation was used to obtain extracts of different molecular weights, and their compositional differences were compared using liquid chromatography–mass spectrometry (LC/MS). POEM contained more proteins and polysaccharides, whereas POES contained more organic acids and alkaloids. These differences in composition were directly responsible for the different degrees of remission of the alleviated UC in model mice. POEM may alleviate UC by regulating the antioxidant capacity and the gut microbiota, whereas the major alleviatory effect of POES was primarily related to the regulation of antioxidant capacity. The POEM and POES effects identified in this study provide a theoretical basis for the development of purslane as a functional food.

## 1. Introduction

Ulcerative colitis (UC) is a type of inflammatory bowel disease (IBD) that is characterized by inflammation, changes in the microbiota, a decrease in thickness of the mucous membrane, and rupture of the intestinal barrier [1]. With the development of modern society, the prevalence of ulcerative colitis is increasing worldwide. The incidence rate of countries located in the western and northern regions is higher than that of countries in the eastern regions, but the recognition of ulcerative colitis is increasing in Asia, the Middle East, and South America [2]. Although the pathogenesis of UC remains unclear, its occurrence is closely linked to genetics, gut microbiota, immune response, diet, and environmental factors [3]. Many drug types have been developed to treat this disease. However, 15% of patients have failed to receive effective medical treatment or were secondary to chronic colitis, subsequently requiring surgical treatment [4]. Therefore, the development of safer and more effective methods for UC prevention and treatment warrants further study.

Natural products, such as medicinal plants and plant-derived bioactive/nutritional compounds, have shown therapeutic effects against UC [5]. In studies, the extract of *Alpinia hainanensis* rhizome containing diarylheptanoids, flavonoids, phenolics, and their hybrid mixtures has been found to reduce the inflammatory response of UC [6]. The extract of *Bruguiera gymnorrhiza* leaves shows its anti-inflammatory and anti-oxidative ability in the treatment of UC [7]. The therapeutic mechanisms underlying extracts of medicinal plants may be related to improving immune dysfunction and regulating the balance of inflammatory factors and antioxidants in patients with UC [3]. Purslane (*Portulaca oleracea* L.) is a common wild vegetable that can alleviate many diseases [8]. It contains many types of active substances, including alkaloids, terpenoids, flavonoids, and organic acids [9]. The few known studies on UC treatment using purslane aqueous extract have mainly focused on autophagy regulation and anti-inflammatory effects [10,11,12]. And there are limited studies analyzing the composition of the aqueous extract. The purslane aqueous extract contains not only small molecules but also large molecules such as polysaccharides and proteins. However, the role of these mixed components in UC remains unclear.

The gut microbiota plays an extremely important role in the treatment of UC, which is not only related to inflammation, but also affects the intestinal barrier and oxidative stress. The gut barrier and microbiota engage in two-way communication. The intestinal environment provides a habitat and nutrients for the microbes. In turn, intestinal flora regulate the secretion of mucin in the mucus layer [13]. Lipopolysaccharides (LPS) are the surface glycolipids of bacteria. When intestinal leakage occurs due to intestinal barrier breakdown, LPS enter the body to induce endotoxemia and accelerate the inflammatory response, which is driven by the immune system and the gut microbiota [14]. LPS can promote the release of more reactive oxygen species (ROS) from mouse macrophages, and ROS can affect the maintenance of intestinal stem cells (ISC) in the intestinal barrier [15]. Moreover, excessive ROS accumulation can cause protein denaturation, lipid peroxidation, DNA damage, and apoptosis [16]. A feature of ulcerative colitis is a change in ‘healthy’ microbiota, and a concurrent reduction in short-chain fatty acids (SCFAs) [17]. SCFAs are the important fuel for intestinal epithelial cells, which enhance intestinal barrier function and immunomodulatory function [18].

Membrane separation technology was used to divide purslane aqueous extract into four main components to further understand its role in UC. The polysaccharide and protein contents and antioxidant capacity of each component were compared. The effects of purslane extracts on the expression of cytokines, the antioxidant enzymes’ content, the intestinal barrier, and the gut microbiota were investigated using a dextran sulfate sodium (DSS)-induced acute colitis mouse model. Different effects of purslane macromolecular (POEM) and small molecular extracts (POES) on UC were found, providing a theoretical basis for the development of purslane as a functional food.

## 2. Materials and Methods

### 2.1. Materials

Fresh purslane was purchased from Jining Guande Trading Co., Ltd. (Jining, China). DSS (MW:36000–50000) was purchased from Yeasen Biotechnology Co., Ltd. (Shanghai, China); sulfasalazine (SASP) was purchased from Shanghai Aladdin biochemical technology Co., Ltd. (Shanghai, China); IL-6, IL-10, and TNF-α ELISA kits were purchased from Dakewe Bio-engineering Co., Ltd. (Shenzhen, China); the BCA protein assay kit was purchased from Beijing Bioss Biotechnology Co., Ltd. (Beijing, China); hematoxylin and eosin (HE) and alcian blue/periodic acid-Schiff (AB/PAS) staining kits were purchased from Beijing Solarbio Science & Technology Co., Ltd. (Beijing, China); zona occludens-1 (ZO-1) rabbit polyclonal antibodies (pAb) and claudin-1 rabbit pAb were purchased from ABclonal (Wuhan, China); occludin polyclonal antibody was purchased from Proteintech Group, Inc (Wuhan, China); the superoxide dismutase (SOD), catalase (CAT), glutathione peroxidase (GSH-px), myeloperoxidase (MPO), and malondialdehyde (MDA) assay kits were purchased from Nanjing Jian-Cheng Bioengineering Institute Co., Ltd. (Nanjing, China). Short-chain fatty acids (lactic acid, acetic acid, propionic acid, and butyric acid) and antioxidant capacity in vitro-related reagents (such as 2,2′-Azino-bis (3-ethylbenzothiazoline-6-sulfonic acid), diammonium salt (ABTS), 1,1-Diphenyl-2-picrylhydrazine (DPPH), and 2,4,6-Tri(2-pyridyl)-s-triazine (TPTZ)) were purchased from Sigma-Aldrich (St Louis, MO, USA), and all other chemical reagents used were of analytical grade.

### 2.2. Preparation of Purslane Extraction

**Figure 1:**
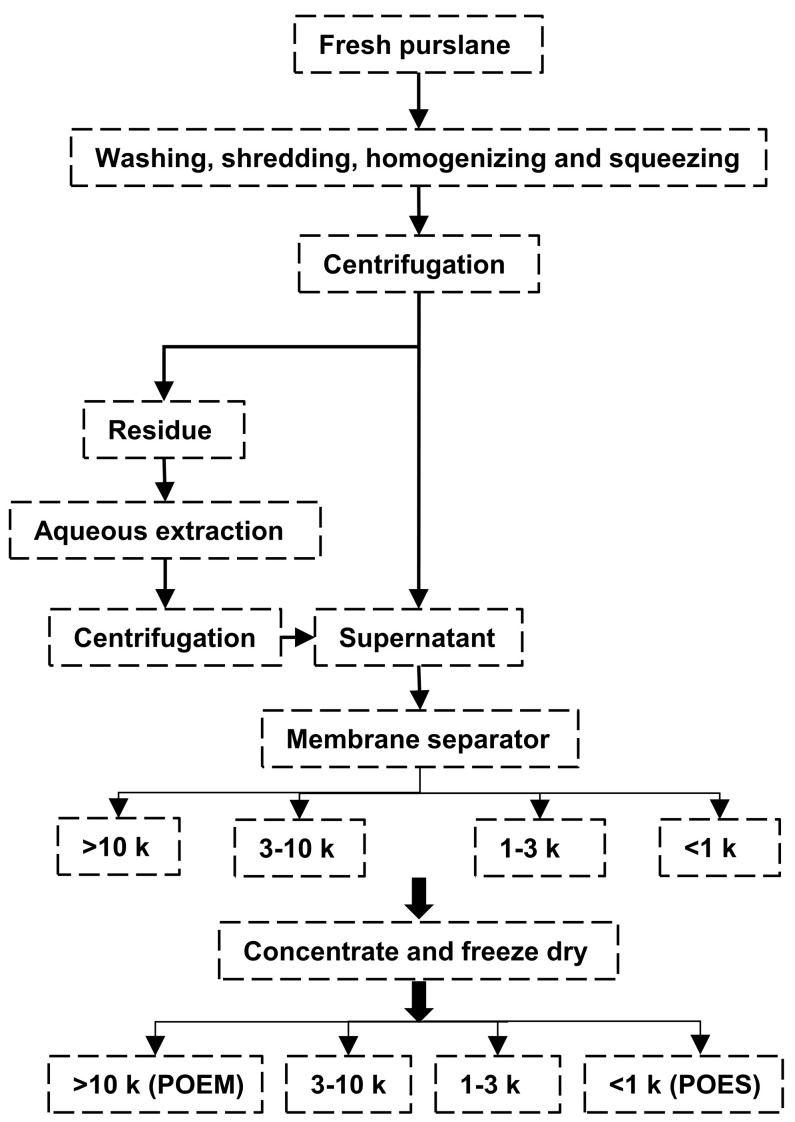
Flow chart for extraction of aqueous extracts of fresh purslane. The >10 k molecular weight of purslane aqueous extract was defined as POEM, and the <1 k molecular weight of purslane aqueous extract was defined as POES.

Two kilograms of fresh purslane was washed, cut into small sections, homogenized, and at 25 °C pressed to release its juice (10 L water added, colloid grinding) at 4000 rpm for 10 min, and the supernatant was collected. Then, 4 L of water was added to the sediment at 80 °C for 1 h. At 25 °C, the samples were centrifuged at 4000 rpm for 10 min, and the supernatant was collected. This process was repeated three times and the supernatants were mixed. Components with molecular weights > 10 kDa, 3–10 kDa, and 1–3 kDa and <1 kDa were separated using a small membrane separator (instrument type: BONA-GM-18 and membrane numbers: BP122772, BP121753, and BP122433 were purchased from Shandong Bona Biological Technology Group Co., Ltd. (Shandong, China)). The separated extracts were concentrated through rotary evaporation at a temperature <60 °C and freeze-dried to obtain 19.4 g of POEM (>10 kDa) and 24.78 g of POES (<1 kDa), which were stored at −20 °C. The extraction process is shown in Figure 1.

### 2.3. Analysis of Components and Antioxidant Capacity in Purslane Extracts

The polysaccharide content was determined using the phenol–sulfuric acid method [19], whereas the protein content was determined using a BCA kit. The in vitro antioxidant potential of purslane aqueous extracts was determined through DPPH, ABTS, hydroxyl radical scavenging capacity, and ferric reducing antioxidant power (FRAP) assays, as previously described [20]. The antioxidant capacity results of DPPH, ABTS, and hydroxyl radical scavenging capacity were expressed by the scavenging rate. The results of three repeated measurements were expressed as the antioxidant capacity corresponding to each 0.1 mg/mL sample, and Vitamin E (VE) was used as the positive control.

### 2.4. Liquid Chromatography–Mass Spectrometry (LC/MS) Analysis of the Components of Purslane Extracts

Thermo Scientific Vanquish was used to analyze the extract components, and the chromatographic column was an ACQUITY UPLC ^®^ BEH C18 (1.7 µm, 2.1 × 50 mm) coupled with mass spectrometer detector Q Executive. Conditions: solution A was H_2_O containing 0.1% formic acid (FA); solution B was acetonitrile containing 0.1% FA; the column temperature was 40 °C; the flow rate was 0.2 mL/min; and loading volume was 1 μL. The elution conditions, mass spectrum conditions, and mass spectrum collection method are presented in Tables S1, S2, and S3, respectively. Substances in the aqueous extracts were characterized according to ChemSpider, mzCloud, and mzVault. The LC/MS data screening is to take POEM as the reference, meet mzCloud score ≥ 70 points, and the top 50 peak area (the sum of the top 50 peak areas accounts for more than 70% of the total peak area), and select the other three groups based on this standard and peak area. After filtering, the comparison results of the other two databases should be satisfied at the same time.

### 2.5. Scanning Electron Microscope Observation

Scanning electron microscopy (Zeiss sigma 300, Oberkochen, Germany) was used to observe the microstructures of POEM and POES. The samples were sprayed with gold and collected under vacuum at different magnifications.

### 2.6. Animals and the Experimental Design

Fifty specific pathogen-free (SPF) C57BL/6 male mice (8 weeks old, 20 ± 2 g) were purchased from Liaoning Changsheng Biotechnology Co., Ltd. (Liaoning, China) (License No.: SYXK (Liao) 2020-0001). All mice were randomly divided into five groups and transferred to plastic cages in an air-conditioned chamber (temperature = 24–25 °C, humidity = 60–65%) under a 12 h light/dark cycle. The animal experiments were registered and approved by the Experimental Animal Welfare Ethics Committee of Jilin University. The ethics review number was 2021SY0716.

The experimental grouping and specific operation are as follows:

(1) CON group: Drinking water was given to the mice for Days 1–8 of intervention. Then, the same volume of phosphate-buffered saline (PBS) was administered by gavage during the experiment.
(2) DSS group: Drinking water containing 4% DSS was given to the mice for Days 1–8 of intervention. Then, the same volume of PBS was administered by gavage during the experiment.
(3) POEM group: Drinking water containing 4% DSS was given to the mice for Days 1–8 of intervention. Then, 400 mg/kg/day POEM was administered by gavage during the experiment.
(4) POES group: Drinking water containing 4% DSS was given to the mice for Days 1–8 of intervention. Then, 400 mg/kg/day POES was administered by gavage during the experiment.
(5) SASP group: Drinking water containing 4% DSS was given to the mice for Days 1–8 of intervention. Then, 200 mg/kg/day SASP was administered by gavage during the experiment.

According to previous research, the SASP-positive drug was administered at 200 mg/kg/day [21]. The dosage of POES and POEM was based on the previous study of the aqueous extract of purslane in our laboratory. The calculation method was based on the consumption and extraction conversion rate of the aqueous extract of purslane. The mice were weighed daily. At the end of the experiment, all mice were euthanized by carbon dioxide inhalation. The contents were collected, and the colon length and spleen weight were measured.

### 2.7. Assessment of Disease Activity Index (DAI)

Body weight, fecal properties, and degree of blood in the stool were recorded daily for each group of mice during the experiment. The slightly modified DAI calculation was the sum of the scores for all the parameters [22].

### 2.8. Assessment of Oxidative Stress Markers and Inflammatory Cytokines

Colon tissue was homogenized in normal saline, and the SOD, CAT, GSH-px, MPO, and MDA contents in colons were measured using kits according to the manufacturer’s instructions. Next, the serum was diluted with normal saline, and the cytokines in the serum were measured using an ELISA kit according to the manufacturer’s instructions.

### 2.9. Histological and Immunohistochemical Analyses

After euthanizing the mice, the colon contents were removed, and the 1 cm colon was immediately fixed with 10% formalin buffer solution, dehydrated, and embedded in paraffin. Sections (5 μM thick) were stained with HE and AB/PAS, and the specific detection method was performed according to the kit manufacturer’s instructions. For immunohistochemical analysis of tight junction proteins, paraffin sections were routinely dewaxed to water, placed in a buffer solution containing 0.01 M citric acid (pH 6.0) and repaired under high pressure. Endogenous peroxidase activity was then eliminated. The serum was sealed and incubated at 25 °C, and the primary antibody (dilution ratio:1:2000) was added overnight. Biotin-labeled secondary antibodies were added after PBS flushing. Then, enzyme labeling was performed after PBS washing. Finally, 3,3-N-Diaminobenzidine tetrahydrochloride color-developing agent, hematoxylin, gradient alcohol, and xylene were added, and then colon sections were sealed with neutral glue.

### 2.10. Analysis of Gut Microbiota

Colonic contents were collected from each group of mice for intestinal microbiota analysis. Total genomic DNA was extracted from the samples using the cetyltrimethylammonium bromide (CTAB) method. DNA was diluted to 1 ng/µL using sterile water in a concentration-dependent manner. Then, 16S rRNA genes of distinct regions (16S V3-V4, 341F (5′-CCTAYGGGRBGCASCAG-3′) and 806R (5′-GGACTACNNGGGTATCTAAT-3′)) were amplified using specific primers with the barcode. The PCR products were mixed in equal proportions, and the Qiagen gel extraction kit (Qiagen, Hilden, Germany) was used to purify the mixed PCR products. The library was established according to the manufacturer’s instructions and its quality was evaluated. Finally, it was sequenced on an Illumina NovaSeq platform. Denoising was performed with the DADA2 or deblur module in the QIIME2 software (Version QIIME2-202006) to obtain initial amplicon sequence variants (ASVs) (default: DADA2). Then, ASVs with an abundance less than 5 were filtered out [23]. Finally, QIIME2 software was used to compare and analyze the data.

### 2.11. Determination of SCFAs Using High-Performance Liquid Chromatography (HPLC)

Here, 3-Nitrophenylhydrazine hydrochloride (3-NPH·HCl) was dissolved in methanol to a final concentration of 20 mM. 1-Ethyl-3-(3-dimethylaminopropyl) carbodiimide hydrochloride (1-EDC·HCl) was dissolved in a 3% pyridine methanol solution to obtain a final concentration of 250 mM. Then, the colon contents were accurately weighed. The weight was recorded, ultrapure water was added to adjust to 0.2 g/mL, and a vortex mixer was used to fully mix the samples. The SCFA contents were determined through HPLC after derivatization [24].

### 2.12. Statistical Analysis

GraphPad Prism 7 was used to analyze the data and corresponding statistics. All data are expressed as mean ± SD. Significant differences were determined by one-way analysis of variance (ANOVA) using IBM SPSS Statistics 22, complemented by Tukey’s test or Duncan’s test. The differences in the ratio of Firmicutes to Bacteroidota (F/B) was determined using Duncan’s test, and Tukey’s test was used for the rest. The differences between the POEM and POES groups at family and genus levels were measured using the *t*-test. *p* < 0.05 was set as the significance level. Origin 2021 was used to analyze the representative curves of SCFAs. The graphical abstract was created by Figdraw (www.figdraw.com (accessed on 30 April 2023)).

## 3. Results

### 3.1. Preparation, Composition Analysis, and Antioxidant Capacity Determination of Aqueous Extracts from Purslane with Different Molecular Weight Ranges

**Figure 2:**
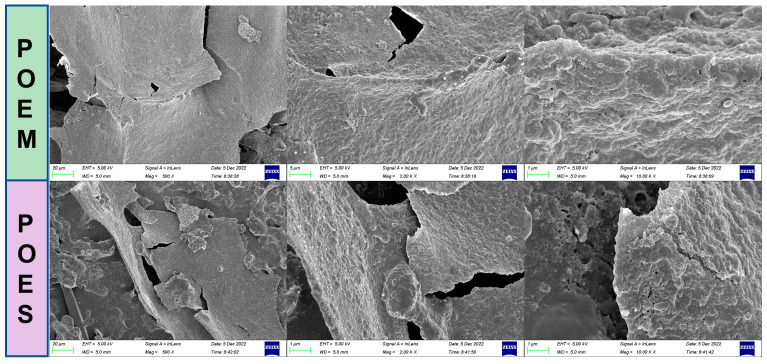
The micro differences between purslane macromolecular aqueous extract (POEM) and purslane small molecular aqueous extract (POES), as observed through the ZEISS 300 microscope at 500, 2 k and 10 k magnification.

The extract was separated using 10 kDa, 3 kDa, and 1 kDa membrane separators to obtain components with different molecular weight ranges. As shown in Table 1, comparing the contents of the main components in the extracts revealed that the polysaccharide and protein contents occupy a high proportion in POEM, indicating a high concentration of these molecules in the POEM component after membrane separation. Moreover, LC/MS demonstrated that the extracts contained abundant small molecules, such as organic acids, amino acids, nucleotide-related substances, and alkaloids. However, the types of small molecules in the aqueous extracts decreased with an increase in the molecular weight (>10 kDa) of the aqueous extracts (Table 2, Figure S1). Most of the small molecules retained were components of proteins and genes. In this study, POES and POEM showed good antioxidant capacity (Table S4). The microstructure of POEM was observed using an electron microscope. As shown in Figure 2, this microstructure is relatively dense, the surface is smooth, and the POES are fragmented. Small holes could be seen at 10 k magnification on the surface of POES. However, POEM was relatively compact.

### 3.2. POEM and POES Alleviated the Pathological Features of DSS-Induced UC in Mice

**Figure 3:**
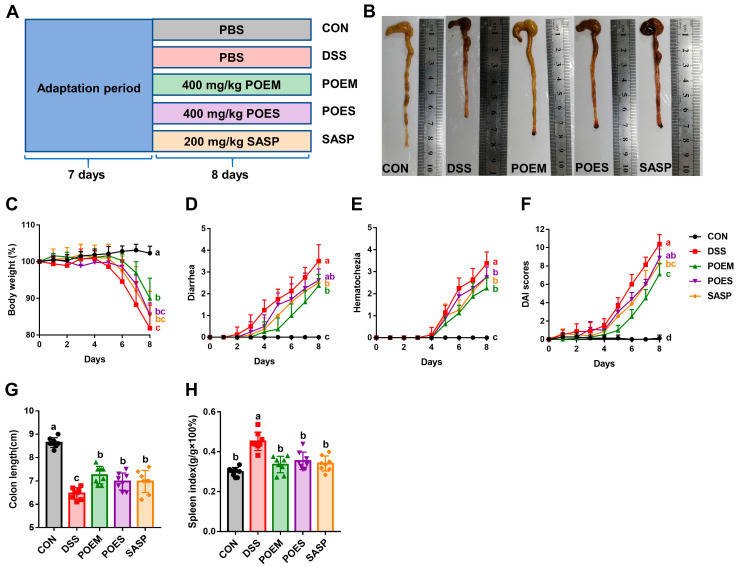
Effects of purslane aqueous extracts on disease symptoms of dextran sulfate sodium (DSS)-induced ulcerative colitis (UC) in mice. (**A**) Animal experimental design, (**B**) representative colon images, (**C**) weight, (**D**) diarrhea score, (**E**) blood in stool score and (**F**) disease activity index (DAI) score of each group, (**G**) colon length of each group, (**H**) spleen index. The data are expressed as mean ± SD (*n* = 8). Data with different letters are significantly different (*p* < 0.05).

POEM and POES were selected based on the extract content for subsequent animal experiments. The animal experimental scheme is shown in Figure 3A. After the one-week adaptation period, all groups except the CON group were given 4% DSS drinking water. The POEM, POES, and SASP groups were given gavage of 400 mg/kg POEM, 400 mg/kg POES, and 200 mg/kg SASP, respectively. In addition, the CON and DSS groups were administered the same volume of PBS. The entire intervention period lasted eight days. Colon length was shorter in the DSS group than in the CON group, and there was a significant difference between the DSS and POEM, POES, and SASP groups. However, colon length did not return to the same level as CON (Figure 3B,G). Weight loss, diarrhea, hematochezia, and DAI scores were considerably increased, which is a typical feature of UC. Intervention with aqueous extracts and SASP reduced weight loss in mice. Similarly, diarrhea, hematochezia, and DAI scores were reduced (Figure 3C–F). Here, the DSS-induced UC mouse model aggravated the spleen index and burdened the spleen. However, purslane intervention groups and SASP groups restored it to the level of the CON group (Figure 3H). Both aqueous extracts of purslane showed improvement in UC.

### 3.3. POEM and POES Regulate Antioxidant Enzymes Levels and Reduce Inflammation

**Figure 4:**
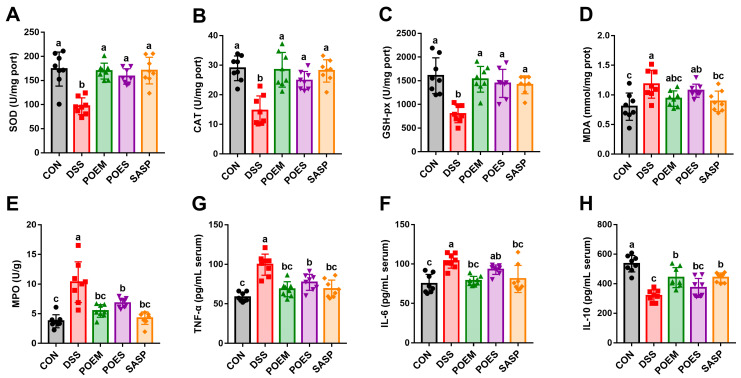
Effects of purslane aqueous extracts on oxidative stress and inflammation. Effects of purslane aqueous extracts on (**A**) superoxide dismutase (SOD), (**B**) catalase (CAT), (**C**) glutathione peroxidase (GSH-px), (**D**) malondialdehyde (MDA), and (**E**) myeloperoxidase (MPO) in the colon of mice. Effects of purslane aqueous extracts on (**F**) interleukin-6 (IL-6), (**G**) tumor necrosis factor alpha (TNF-α), and (**H**) interleukin-10 (IL-10) in mouse serum. Data are expressed as mean ± SD (*n* = 8). Data with different letters are significantly different (*p* < 0.05).

DSS substantially reduced the antioxidant enzyme contents and increased the contents of MDA, a product of lipid peroxidation (Figure 4A–D). POEM, POES, and SASP can considerably restore antioxidant enzyme contents, with a tendency to reduce MDA. Purslane aqueous extracts and positive drugs can effectively reduce the inflammatory state of UC by reducing the contents of pro-inflammatory cytokines IL-6 and TNF-α and MPO (a marker of neutrophil infiltration). Furthermore, they can increase the contents of the anti-inflammatory factor IL-10 (Figure 4E–H). POEM had the most considerable effect on the reduction of pro-inflammatory factors, and its recovered contents had no significant difference with those of the CON group (Figure 4E,F). These results suggest that POEM and POES can regulate oxidative stress and inhibit inflammation.

### 3.4. POEM and POES Protect Colon Integrity and Increase the Tight Junction

**Figure 5:**
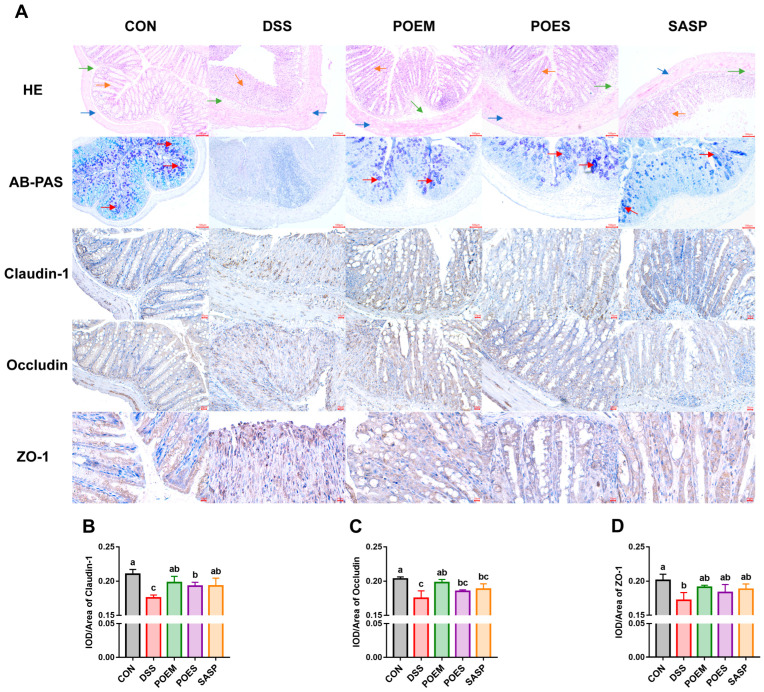
Effects of purslane aqueous extracts on the intestinal barrier of mice with dextran sulfate sodium (DSS)-induced ulcerative colitis (UC). (**A**) hematoxylin and eosin (HE) staining (The scale bar is 100 μm), and alcian blue/periodic acid-Schiff (AB/PAS) staining (The scale bar is 100 μm), and immunohistochemical analysis of the tight junction proteins of colon tissue (The scale bar is 10 μm). The blue, green, orange, and red arrows point to the muscular, submucosal, and mucosal layers, as well as mucin stained by AB/PAS, respectively. (**B**) Claudin-1 expression in colon tissue. (**C**) Occludin expression in colon tissue. (**D**) ZO-1 expression in colon tissue. Data are expressed as mean ± SD (*n* = 4). Data with different letters are significantly different (*p* < 0.05).

The intact and closely connected epithelial cells can form a barrier to prevent the leakage of harmful substances, which plays a crucial role in intestinal immunity [25]. HE staining showed that the CON had a complete colon structure. Moreover, the mucosal and submucosal layers were destroyed in the DSS group, with extensive inflammation observed (Figure 5A). AB/PAS staining is typically utilized to show the state of mucin in the colon. Goblet cells in the CON group normally secreted mucin, and the mucin distribution could be seen in the colon. In contrast, DSS destroyed goblet cells and reduced mucin secretion. The POEM, POES and SASP groups protected the integrity of colon tissue, retained the crypt structure, reduced the damage to goblet cells, restored mucin secretion, and reduced inflammation (Figure 5A). Immunohistochemical analyses revealed that the expression of tight junction-associated proteins claudin-1, occludin, and ZO-1 were decreased in the DSS group. The tight junction-associated proteins in the purslane intervention groups and SASP groups recovered after intervention (Figure 5B–D).

### 3.5. POEM and POES Regulated the Intestinal Microbiota Composition

**Figure 6:**
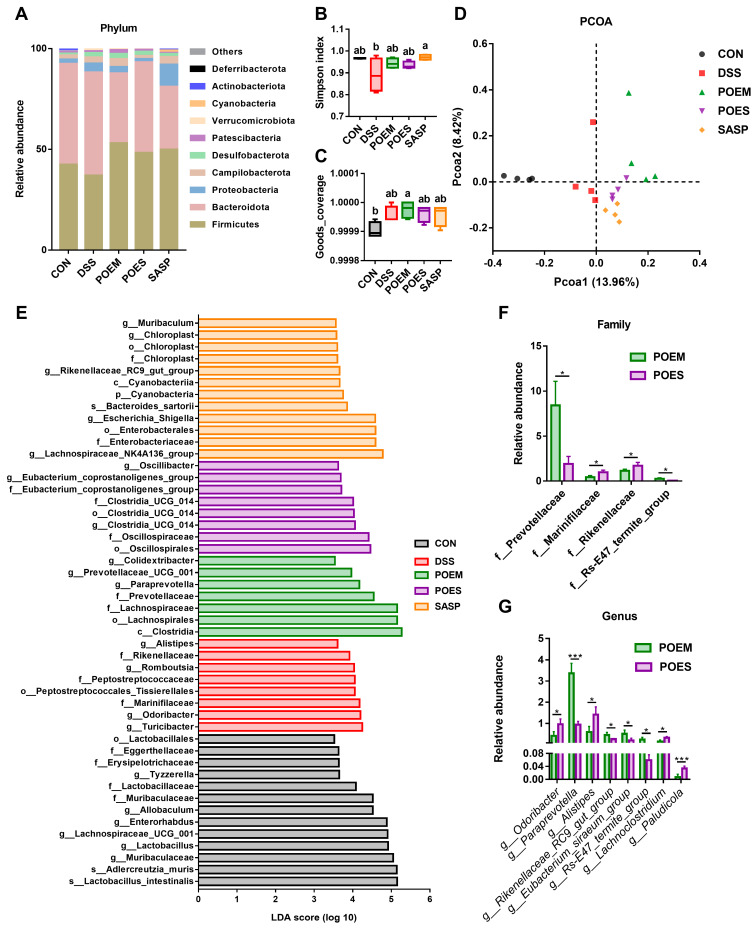
Effects of purslane extracts on the gut microbiota of mice with dextran sulfate sodium (DSS)-induced ulcerative colitis (UC). (**A**) The distribution of the intestinal microbiota at the phylum level. (**B**) Simpson index. (**C**) Good’s coverage index. (**D**) Principal coordinate analysis. (**E**) Linear discriminant analysis. (**F**,**G**) *t*-test for determination of the differences between the POEM and POES groups at family and genus levels (*n* = 4). Data with different letters are significantly different (*p* < 0.05). In the results of *t*-test, * *p* < 0.05; *** *p* < 0.005.

Recognition of the importance of the intestinal microbiota in IBD is growing rapidly. Controlling the intestinal microbiota through dietary intervention, prebiotic and probiotic compounds, and fecal microbiota transplantation may expand the therapeutic prospect of UC [26]. In this study, there were the same 315 ASVs in the five groups. Different interventions changed the intestinal microorganisms, and specific ASVs appeared. A total of 150, 162, 175, 215, and 278 ASVs appeared only in the DSS, CON, POEM, POES, and SASP groups, respectively (Figure S2A). Figure 6A shows the proportion of major phylum levels in previous groups. The abundance of Firmicutes decreased after DSS intervention, whereas that of Bacteroidetes and Proteobacteria increased. POEM substantially recovered the F/B ratio after the intervention (Figure S2B). In addition, there were differences in family and genus abundances among the groups (Figure S2D,E). The Simpson index decreased after DSS intervention, showing a significant difference from the SASP group. The intervention groups showed a recovery trend but did not reach a significant difference (Figure 6B). Good’s coverage and the Simpson indices showed α-diversity. Good’s coverage index showed that the sequencing depth of each group was sufficient (Figure 6C). Principal coordinate analysis (PCoA) and nonmetric multidimensional scaling (NMDS) were used to show β-diversity. There was a distance between the sample distribution of the DSS group and that of the other groups (Figure 6D and Figure S2C). Linear discriminant analysis (LDA) was used to analyze different bacteria between groups to find different gut microflora (Figure 6E). The contents of Marinifilaceae, Rikenellaceae, and Peptostreptococachaceae, most of which are harmful to the body, increased in the DSS group. Contrastingly, the POEM group showed an increased abundance of *Paraprevotella*, *Prevotellacee_UCG-001*, and *Colidextribacter*. A *t*-test comparison of POES and POEM at the family and genus levels revealed that the gut microbiota of the POEM group was healthier than that of the POES group (Figure 6F,G).

### 3.6. POEM and POES Affected SCFA Contents in the Intestinal Contents of Mice

**Figure 7:**
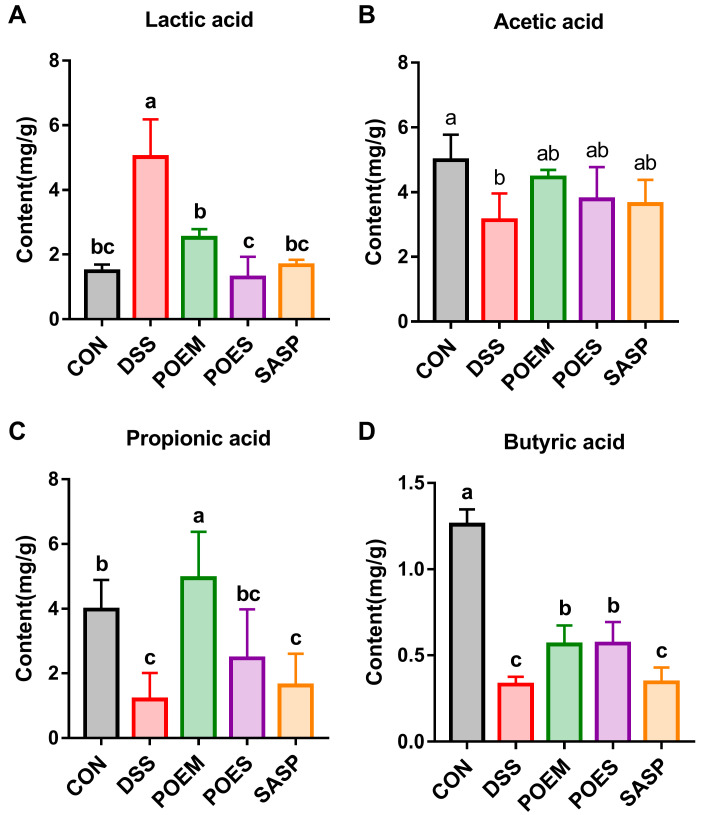
Effects of purslane extracts on contents of short-chain fatty acids in the colon contents of mice in each group: (**A**) lactic acid, (**B**) acetic acid, (**C**) propionic acid, and (**D**) butyric acid. The data are expressed as mean ± standard deviation (*n* = 5). Data with different letters are significantly different (*p* < 0.05).

SCFAs play a key role in the pathogenesis of IBD. SCFAs are beneficial metabolites produced by intestinal flora and play an important role in maintaining intestinal homeostasis. In this study, we detected the SCFA contents in the colon. The contents of acetic acid, propionic acid, and butyric acid in the DSS group decreased, but that of lactic acid increased. This phenomenon could be reversed after POES and POEM were administered (Figure 7A–D and Figure S3).

### 3.7. Correlation between the Main Factors of UC Development and the Gut Microflora

**Figure 8:**
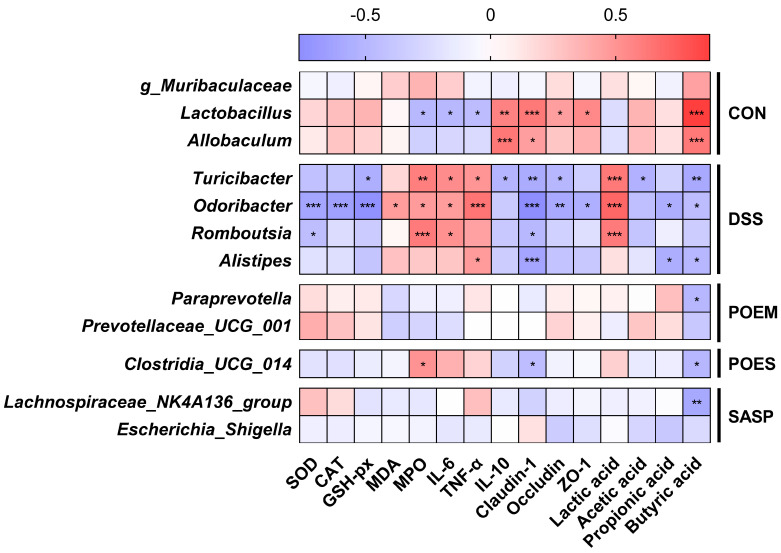
Correlation analysis for ulcerative colitis (UC)-related indicators and the gut microbiota. A heatmap for correlation, as calculated through the Spearman correlation coefficients. Negative correlation (blue) and positive correlation (red) were expressed by color intensity. * *p* < 0.05, ** *p* < 0.01, *** *p* < 0.001, significant correlations.

Pathological symptoms of UC, inflammatory factors, antioxidant enzymes, tight junction proteins expression, and short-chain fatty acids were mostly significantly correlated (Figure S4). This shows that these indicators are related and affect each other. To understand the status of gut microflora in the development and treatment of UC, the Spearman correlation coefficient between indicators related to UC and the gut microflora (the relative abundance of bacteria that were in the top 20 and the differences in bacteria satisfied linear discriminant analysis) were calculated (Figure 8). The significantly enriched *Turiciber*, *Odoribactor*, *Rombousia*, and *Alistipes* in the DSS group were negatively correlated with antioxidant enzymes, anti-inflammatory factors, tight junction proteins, and SCFAs (acetic acid, propionic acid, and butyric acid). However, they were positively correlated with pro-inflammatory factors and lactic acid. In contrast, the significantly enriched *Lactobacillus* and *Allobaculum* in the CON group were significantly negatively correlated with the factors of inflammation development (*Lactobacillus)* and significantly positively correlated with tight junction proteins and SCFAs (butyric acid). Here, *g_Clostridia_UCG_014*, which was significantly enriched in the POES group, was significantly positively correlated with MPO and negatively correlated with claudin-1 and butyrate. The same trend was observed in the microbial enrichment of the DSS group. The correlation trend between POEM-enriched *Paraprevotella* and *Prevotellaceae_UCG_001* and their indicators is similar to that of the CON group. The enriched gut microflora of the DSS group showed a change in the development trend of UC, whereas the gut microflora changed after POEM intervention, showing a healthy trend.

## 4. Discussion

Purslane has various biological properties, such as anti-inflammatory, anti-diabetic, anti-tumor, liver-protective, anti-cancer, antioxidant, and antibacterial activities [27]. Our findings demonstrated that POEM contains a higher proportion of polysaccharides and proteins. Our results also show that POEM also contains small molecules, which may be owing to the complex spatial structure and functional groups of macromolecular materials, as many macromolecular components contain small molecules. A most common example is the extraction process of tea polysaccharides, which contain tea polyphenols that are not removed during dialysis [28]. The polysaccharides [29] in purslane have excellent antioxidant capacity, which may result in the POEM having the same antioxidant capacity as POES in vitro and in vivo. Small molecules in these plant extracts mainly play anti-inflammatory and antioxidant roles in alleviating UC. For example, ferulic acid is a phenolic acid widely found in plants. It has various biological activities, particularly in oxidative stress and inflammation. Ferulic acid can inhibit the PI3K/AKT pathway and ROS production [30]. Similarly, azelaic acid is a dicarboxylic acid with rich biological and therapeutic properties (anti-inflammatory, antioxidant, anti-keratosis, anti-microbial properties) [31]. Uridine can reduce the levels of pro-inflammatory cytokines IL-6, IL-1β, and TNF as well as colon mRNA expression. In addition, it can inhibit neutrophil infiltration and the NF-κB signaling pathway in mice with UC [32]. Meanwhile, other small molecules (citric acid [33], guanine [34], uridine [35], 2’-deoxyadenosine [36], adenosine [37], trigonelline [38], L-phenylalanine [39], and L-tryptophan [40]) in POEM and POES have been reported to have direct or indirect antioxidant capabilities or contributions. Macromolecules can regulate the intestinal flora and restore gut homeostasis following DSS damage. The *Scutellaria baicalensis* Georgi polysaccharide improved DSS-induced UC by improving intestinal barrier function and modulating gut microbiota. This included increasing the abundance of Firmicutes, *Bifidobacterium*, *Lactobacillus*, and *Roseburia* [25]. Polysaccharides extracted from *Arctium lappa* considerably improve gut microbiota by promoting Proteobacteria, Alcaligenaceae, *Staphylococcus*, and *Bacteroidetes*, which are beneficial for the relief of UC [41]. In addition, *Rheum Tanguticum*, *Codonopsis pilosula*, and other plant polysaccharides can regulate intestinal flora and alleviate UC [42]. In addition to polysaccharides, 5–10% of dietary protein is not absorbed as proteins and peptides when it passes through the ileum and enters the colon. It interacts with gut microbes to contribute a small portion of the SCFA pool [43]. Our results were similar to previous reports; POES had anti-inflammatory and significant antioxidant effects in vivo, and POEM had more obvious effects on the intestinal flora.

SCFAs can inhibit the recruitment of monocytes, macrophages, and neutrophils, which have potential anti-inflammatory effects. Butyrate has been proven to have anti-inflammatory effects on immune cells and intestinal epithelial cells at the intestinal level [44]. The SCFA results also implied differences in the intestinal flora. Regulation of the intestinal flora is extremely important in the remission treatment of UC. Gut microbiota balance is crucial for maintaining the physiological function of the host, which can affect the mucosal barrier function and host immune system [5,45,46]. Our study showed that DSS treatment reduced the Simpson index of intestinal microbes in mice. Moreover, experimental intervention increased the diversity of the gut microbiota. PCoA and NMDS analyses showed that the aqueous extract interventions of purslane could clearly aggregate the gut microbiota. Firmicutes and Bacteroides were the main intestinal microorganisms found in this study. Firmicutes are associated with impaired intestinal barrier integrity and LPS leakage [47]. Furthermore, approximately 50% of the microbial proteins related to UC pathogenesis come from Bacteroides [48]. Therefore, the F/B ratio is considered an important marker of intestinal microbiota dysregulation [49]. In addition, Proteobacteria can cause inflammation and change the intestinal flora, thereby promoting the development of IBD [50]. Here, DSS intervention considerably enriched Marinifilaceae, Rikenellaceae, and Peptostreptococcaceae. Previous studies have reported changes in these three bacterial families in UC [28,51,52]. The POEM group significantly increased the content of beneficial bacteria, resulting in a more symbiotic development of intestinal microorganisms.

To demonstrate the difference between POES and POEM in the treatment of UC from the perspective of intestinal microorganisms, the differences in microbiota between the two groups were compared using a *t*-test. Compared with the POEM group, most of the microbiota significantly enriched in the POES group was also enriched in the DSS group. Moreover, POES significantly enriched *Alistipes* [53], *Odoribacter* [54,55], and *Lachnoclostridium* [56], which are related to UC. Therefore, POEM increases the contents of beneficial bacteria. The enriched bacteria in the POEM group were positively correlated with the antioxidant enzymes occludin and ZO-1, as well as acetic acid, and propionic acid. However, they were negatively correlated with MDA, MPO, and IL-6. In contrast, *Clostridia_UCG_014* enriched in the POES group was negatively correlated with the antioxidant enzymes IL-10, claudin-1, occludin, and ZO-1, as well as acetic acid, and propionic acid, but positively correlated with MPO, IL-6, and TNF-α. Patients with UC have considerably less *Clostridia_UCG_014* than healthy individuals. *Clostridia_UCG_014* is a major source of tryptophan metabolites, and the tryptophan-AHR pathway is important in the regulation of intestinal homeostasis [57]. The two bacteria that were enriched in the POEM group can use proteins and polysaccharides to confer beneficial effects on intestinal homeostasis. *Paraprevotella* strains are effective trypsin-degrading symbiotic bacteria that can help maintain intestinal homeostasis and protect against pathogens [58]. *Prevotellaceae UCG-001* can utilize fibers, produce SCFAs, play an anti-inflammatory role in immune cells, and inhibit the growth of invasive pathogens [59,60].

In conclusion, our results show that although POES and POEM can relieve UC to varying degrees, their effects differ. POES mainly affects oxidative stress, possibly due to the abundance of organic acids and alkaloids. The polysaccharides and proteins contained in POEM can directly interact with intestinal microorganisms and play a more obvious mediating role in intestinal bacteria. However, the current study has some limitations. Firstly, owing to the spatial structure of macromolecules or the formation of bonds with small molecules, membrane separation technology cannot completely separate small molecules from macromolecules. Secondly, this study analyzed the composition of microbes using 16S rRNA gene sequencing. However, its depth could not fully show the gut microbiota at a more detailed classification level. Finally, the role of macromolecular substances, such as polysaccharides and proteins, in the aqueous extract of purslane remains unclear in the treatment of UC. Therefore, it is necessary to further classify and purify the components of POEM and investigate the effects of and mechanism underlying UC alleviation.

## Figures and Tables

**Table 1:** Contents of aqueous extracts from purslane after membrane separation. ^1^ Aqueous extract: aqueous extract of purslane before membrane separation. The data are expressed as mean ± SD (*n* = 3) and were analyzed through one-way ANOVA.

No.	Sample	Weight (g/kg)	Polysaccharide (mg/g)	Protein (mg/g)
1	Aqueous extract ^1^	27.85	117.15 ± 14.30	183.38 ± 21.60
2	>10 kDa (POEM)	9.70	159.87 ± 8.83	269.13 ± 11.50
3	3–10 kDa	0.04	75.49 ± 10.52	173.70 ± 7.27
4	1–3 kDa	0.02	61.89 ± 2.87	136.76 ± 18.53
5	<1 kDa (POES)	12.39	56.94 ± 3.47	120.49 ± 6.64

**Table 2:** LC/MS identification of the compositions of aqueous extracts from purslane at each molecular weight interval.

No.	Classification	<1 kDa (POES)	1–3 kDa	3–10 kDa	>10 kDa (POEM)
1	Organic acids	Citric acid	Citric acid	-	Citric acid
2		Ferulic acid	Ferulic acid	Ferulic acid	-
3		Azelaic acid	Azelaic acid	Azelaic acid	-
4		-	Pantothenic acid	-	-
5		-	-	Isocitric acid	-
6		-	-	D-(-)-Quinic acid	-
7	Nucleotide related substances	Guanine	Guanine	Guanine	Guanine
8		Adenosine	Adenosine	Adenosine	Adenosine
9		Uridine	Uridine	Uridine	-
10		2′-Deoxyadenosine	2′-Deoxyadenosine	2′-Deoxyadenosine	2′-Deoxyadenosine
11	Amino acids	L-Phenylalanine	L-Phenylalanine	L-Phenylalanine	L-Phenylalanine
12		L-Tryptophan	-	-	-
13		-	D-(+)-Tryptophan	-	-
14		-	-	DL-Tryptophan	DL-Tryptophan
15	Alkaloids	Trigonelline	Trigonelline	Trigonelline	-

ChemSpider, mzCloud, and mzVault were used for identification. The data screening took POEM as the reference, meet mzCloud score ≥70 points, and the top 50 peak area substances, and selected the other three groups based on this standard and peak area. After filtering, the comparison results of two other databases should be satisfied at the same time.

## Data Availability

All of the data is contained within the article and the supplementary materials.

## Supplementary Materials

The following supporting information can be downloaded at https://www.mdpi.com/article/10.3390/antiox12071400/s1: Figure S1. LC/MS results of POEM and POES represent graphs; Figure S2. Effects of extracts of purslane on the gut microflora; Figure S3. Representative graphs of short-chain fatty acids (SCFAs); Figure S4. Correlation analysis of UC-related indicators except gut microbiota; Table S1. HPLC gradient elution; Table S2. Mass spectrum condition; Table S3. Mass spectrum acquisition method; Table S4: Antioxidant capacity of various classifications of aqueous extracts of purslane.
