# Supplementary material for: Protective Effects of Different Molecular Weights of Purslane (Portulaca oleracea L.) Aqueous Extract on DSS-Induced Ulcerative Colitis in Mice
Source: Antioxidants (Basel). 2023 Jul 8;12(7):1400. doi: 10.3390/antiox12071400 (PMC10376347; doi:10.3390/antiox12071400)
Supplement: Supplementary file 1 [file antioxidants-12-01400-s001.zip › antioxidants-2440579-supplementary.pdf]

Supplementary material

1. HPLC conditions

1） Instrument type: Thermo Scientific Vanquish Ultra high performance liquid chromatograph

2） Chromatographic column: ACQUITY UPLC® BEH C18, 1.7 μm, 2.1×50 mm

3） Mobile phase: Mobile phase A: H_2O+0.1%FA; Mobile phase B: ACN +0.1%FA;

4） Column temperature: 40℃

5） Flow rate: 0.2 mL/min

6） Loading volume: 1 μL

7） Gradient elution is as follows:

Table S1. HPLC gradient elution

Time (min)	Mobile phase A (%)	Mobile phase B (%)
0.0	95	5
3.5	85	15
6.0	70	30
6.5	70	30
12.0	30	70
12.5	30	70
18.0	0	100
25	0	100
26	95	5
30	95	5

The setting conditions for the mobile phase are shown in the table.

2. Mass spectrum condition:

Table S2. Mass spectrum condition

Equipment	Parameter
Instrument type	quadrupole/electrostatic field orbitrap high resolution mass spectrometry Q Exactive
Ionization Mode	ES+
Capillary Temperature (°C)	320
Spray Voltage (V)	3.7k (+)
Auxiliary Gas heater temperature (°C)	350
Capillary (kV)	3.0
S-lens RF level	60
Sheath Gas flow rate (Arb)	35
Auxiliary Gas flow rate (Arb)	10

The specific parameters of mass spectrum condition setting are shown in the table.′ Arb′ is the unit of flow rate.

3. Mass spectrum acquisition method:

Table S3. Mass spectrum acquisition method

Ionization Mode	Full MS/dd-MS^2 (Top N)
General	
Runtime	0 to 30 min
Polarity	Positive/Negative
Default charge state	2
Full MS	
Resolution	70,000
AGC target	1e^6
Maximum IT	200 ms
Scan range	133-2000 m/z
dd-MS^2	
Resolution	17,500
AGC target	1e^5
Maximum IT	50 ms
Loop count	10
TopN	10
Isolation window	1.5 m/z
（N） CE/stepped nce	20, 40, 60
dd Settings	
Minimun AGC target	8.00e^3
Apex trigger	4 to 8 s
Exclude isotopes	on
Dynamic exclusion	10.0 s

AGC target: Automatic gain control target.

Table S4. Antioxidant capacity of various classifications of aqueous extracts of purslane

No.	sample	DPPH (%)	HRSA (%)	FRAP (Fe^{2+} mmol/mL)	ABTS (%)
**1**	**Aqueous extract** ^{A}	**18.33±1.99** ^{ab}	**59.81±0.04** ^{ab}	**0.1761±0.0288** ^{a}	**13.11±1.32** ^{a}
**2**	**> 10 kDa (POEM)** ^{A}	**20.64±1.58** ^{a}	**64.86±0.07** ^{a}	**0.1328±0.0088** ^{ab}	**12.39±2.44** ^{a}
**3**	**3-10 kDa** ^{A}	**17.81±2.07** ^{ab}	**60.38±0.01** ^{ab}	**0.1000±0.0074** ^{ab}	**12.09±1.15** ^{a}
**4**	**1-3 kDa** ^{A}	**9.27±1.74** ^{c}	**56.48±0.02** ^{ab}	**0.0673±0.0014** ^{b}	**9.53±0.93** ^{a}
**5**	**< 1 kDa (POES)** ^{A}	**13.67±1.37** ^{bc}	**60.40±0.08** ^{ab}	**0.1229±0.0769** ^{ab}	**12.68±1.81** ^{a}
**6**	**VE**	**10.77±2.44** $^{c\,B}$	**51.27±3.14** $^{b\,C}$	**0.0730±0.0279** $^{b\,C}$	**12.48±2.31** $^{a\,C}$

The red capital letters represent the difference in concentration, which is explained as follows *A* 1 mg/mL sample were determined, *B* 1 mg/mL Vitamin E (VE) as positive control, *C* 10 mg/mL VE as positive control. The data were expressed as mean ± SD (n = 3) and were analyzed by one-way ANOVA. The lowercase letters in the same column with different superscripts are statistically significant (p < 0.05).

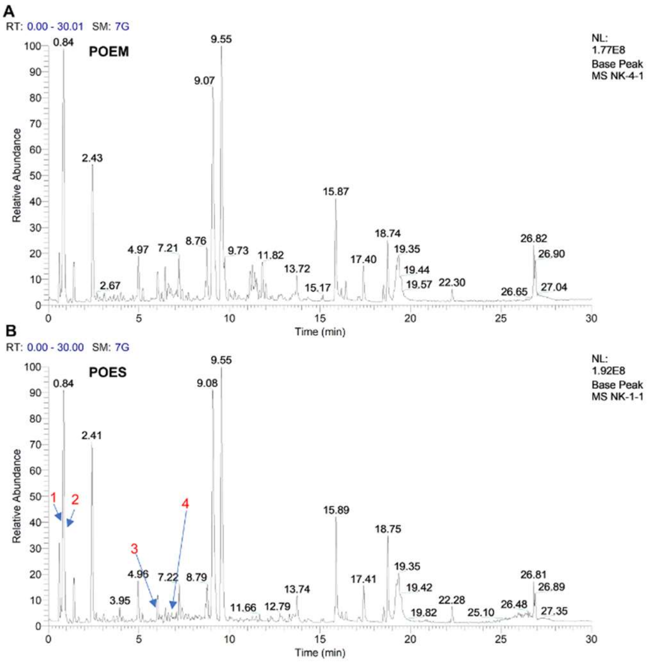

Figure S1. LC/MS results of POEM and POES represent graphs, where A is the picture of POEM results and B is the picture of POES results. The numbers point to material peaks that are inferred based on retention time. '1' stands for trigonelline, '2' for uridine, '3' for ferulic acid, and '4' for azelaic acid.

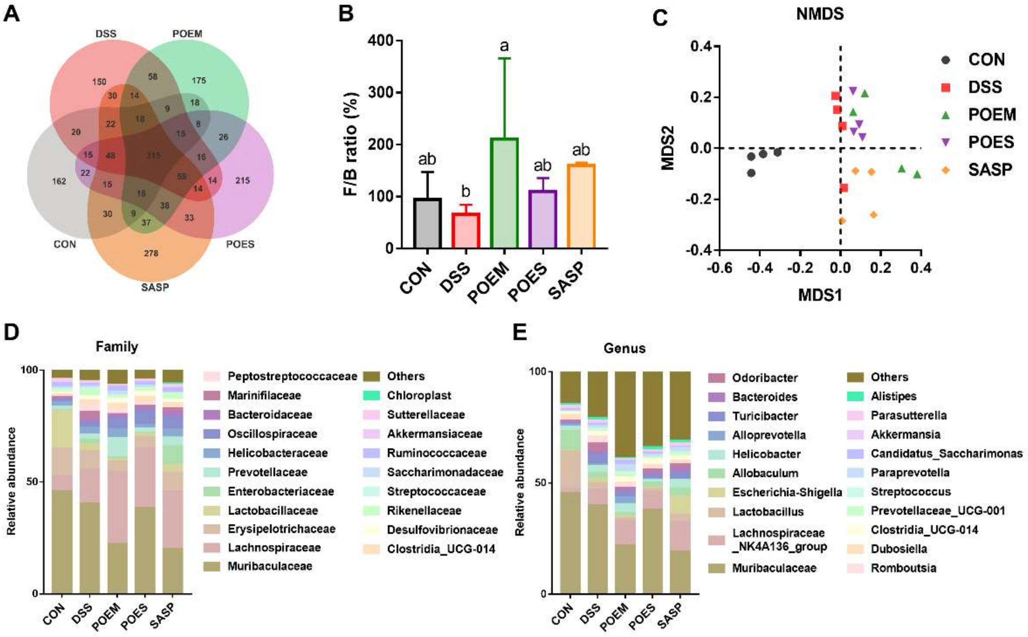

Figure S2. Effects of extracts of purslane on the gut microflora. (A) Petal plots of the gut microbiota in each group. (B) Ratio of Firmicutes to Bacteroidetes. (C) β diversity of gut microbiota was analyzed by Non-metric multidimensional scaling(NMDS). (D) and (E) are the distribution of the intestinal microbiota at the family and genus levels, respectively. The data were expressed as mean ± SD (n = 3 - 4) and were analyzed by one-way ANOVA. Data with different letters are significantly different ($p < 0.05$).

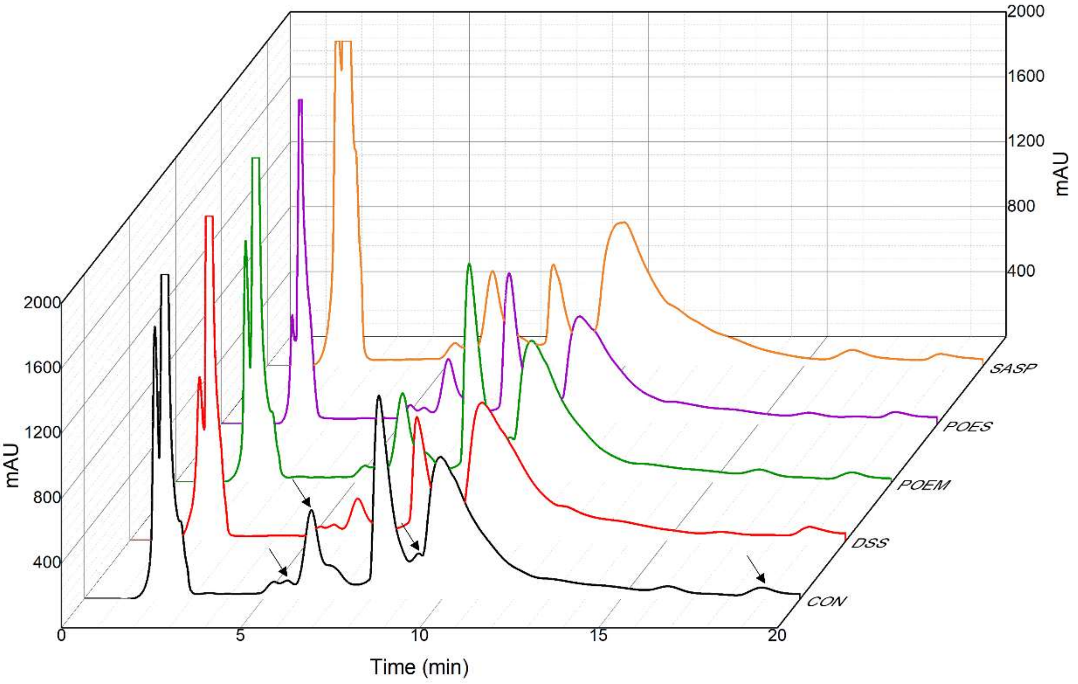

Figure S3. Representative graphs of short-chain fatty acids (SCFAs). Black arrows pointing from left to right represent lactic acid, acetic acid, propionic acid, and butyric acid, respectively.

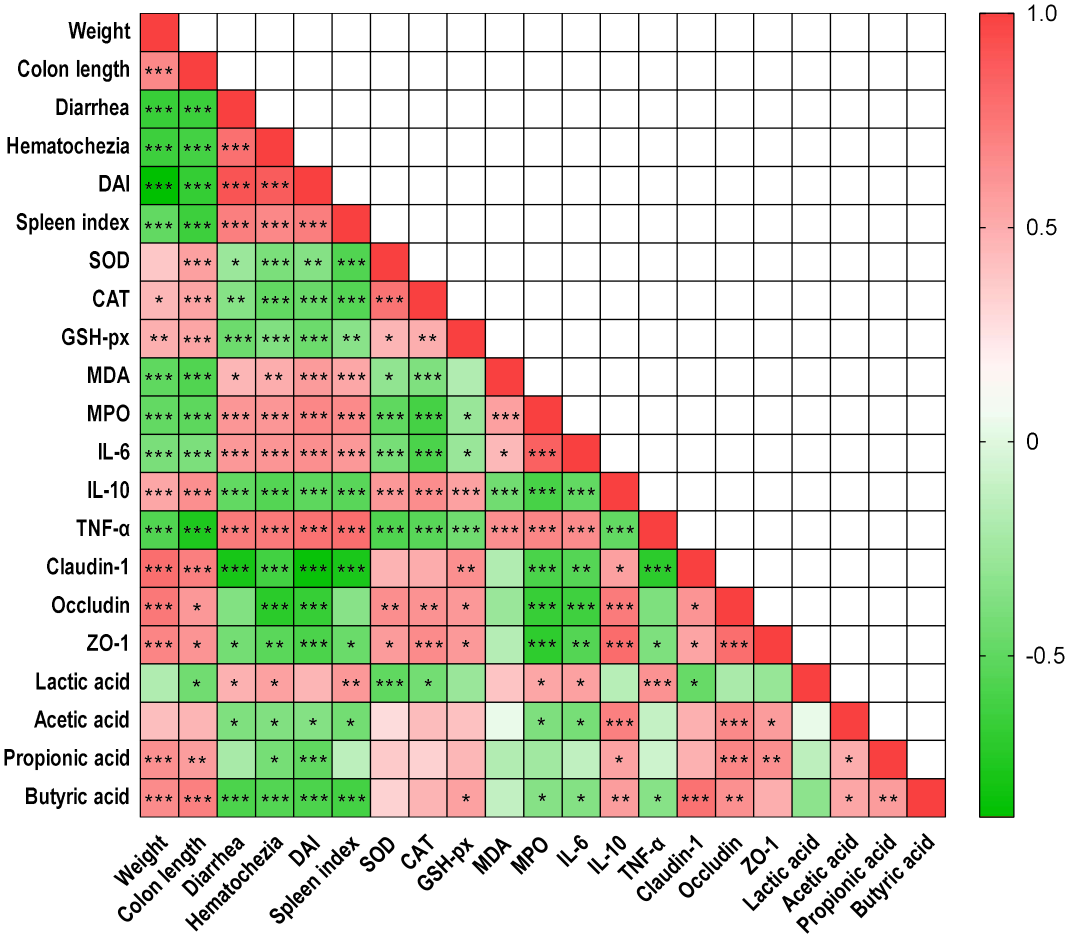

Figure S4. Correlation analysis of UC-related indicators except gut microbiota. Heatmap for correlation which was calculated by the Spearman correlation coefficients. Negative correlation (green) and positive correlation (red) were expressed by color intensity. *p< 0.05, **p< 0.01, ***p< 0.001, significant correlations.
